# Findings of Bone Scintigraphy After Leech Theraphy

**DOI:** 10.4274/Mirt.303

**Published:** 2014-02-05

**Authors:** Sinem Özyurt, Gökhan Koca, Koray Demirel, Aylin Baskın, Meliha Korkmaz

**Affiliations:** 1 Ankara Training and Research Hospital, Department of Nuclear Medicine, Ankara, Turkey

**Keywords:** Leech therapy, hirudo medicinalis, hyperemia, scintigraphy, Tc-99m MDP

## Abstract

In this case report, we present a 70 year old female patient who had recieved Leech therapy (hirudotherapy) on her leg without informing referring physician. In dynamic bone scintigraphy there was increased perfusion and hyperemia in her left ankle and leg, also in late static images moderate increased uptake was seen in soft tissue region and at the fracture site of ankle. We learned that she had Leech therapy applied on her leg, which could explain the increased perfusion and hyperemia in dynamic and blood pool phases of bone scintigraphy because of Leech therapy’s dilatory effects on superficial veins. Leech therapy may lead to an increase in perfusion and hyperemia in blood pool phase of bone scintigraphy, which may cause confusion in differential diagnosis. To our best knowledge this report is the first case that shows the scintigraphic findigs after Leech therapy.

**Conflict of interest:**None declared.

## Abstract (tr)

Bu vaka sunumunda, doktorunun bilgisi olmaksızın bacağına sülük tedavisi (Hirudoterapi) uygulanan 70 yaşındaki bir kadın hasta sunuyoruz. Dinamik kemik sintigrafisinde sol ayak bileğinde ve sol bacakta artmış perfüzyon ve hiperemi, ayrıca geç statik görüntülerde de yumuşak dokuda hafif radyoaktivite tutulumu ile sol ayak bileğindeki kırık bölgesinde tutulum gözlendi. Hastanın bacağına sülük tedavisi aldığını öğrendik, bu da sülük tedavisinin yüzeysel venlere dilatör etkilerinden dolayı, kemik sintigrafisinde dinamik ve kan havuzu fazında perfüzyon artışını ve hiperemiyi açıklıyordu. Hirudoterapi kemik sintigrafisinde perfüzyon artışına ve kan havuzu fazında hiperemiye neden olup, ayırıcı tanıda karışıklığa yol açabilir. Bu rapor bizim bildiğimiz kadarıyla sülük tedavisi sonrası sintigrafik bulguları gösteren ilk vakadır.

## INTRODUCTION

Bone scintigraphy was first performed in 1971 by using Tc-99m labeled diphosphonates (Tc-99m MDP, Tc-99m-HDP) and has taken its place in Nuclear Medicine applications (1). At present, it is one of the most commonly used radionuclide imaging methods. Although the specificity of bone scintigraphy is low, its sensitivity is high, which makes it useful in many pathologic conditions. It is a rapid, cheap and easily accessible imaging method, and after the intravenous injection of radiopharmaceutical, approximately 50% of it, is absorbed by bones. The peak activity of bone is reached at the first hour after injection and the highest target/background activity ratio is seen 6-12 hours later (2). If bone scintigraphy is performed for infection or trauma, multi phase imaging is required. Blood flow and blood pool (activity of soft tissue) images of the suspected area are taken following the radionuclide injection and then late phase images are taken 2-4 hours later. Differentiation of osteomyelitis and soft tissue infection (cellulitis) from each other, evaluation of painful joint prothesis, trauma, bone greft viability and Complex Regional Pain Syndrome (RSD) are all made possible with dynamic (3 or 4 phases) bone scintigraphy (2). If the bone has not previously been affected by pathological conditions, the accuracy of bone scintigraphy for diagnosis of osteomyelitis is high and it is a cost-effective modality with high sensitivity and spesificity (90% and 95%, respectively). However if the bone has been affected previously by a pathological process such as orthopedic surgical procedures, although sensitivity of bone scan remains high, its spesificity falls to 30% (3).

Leech therapy has important therapeutic effects, so it has gained globally greater attention. The saliva of leech contain anesthetic, vasodilator, antiinflamatory, protease inhibitory and anticoagulant substances. During feeding, leeches secrete these active substances into the wound (4). These substances increase arterial flow and lead to hyperemia in bone scintigraphy.

## CASE REPORT

**Figure 1:**
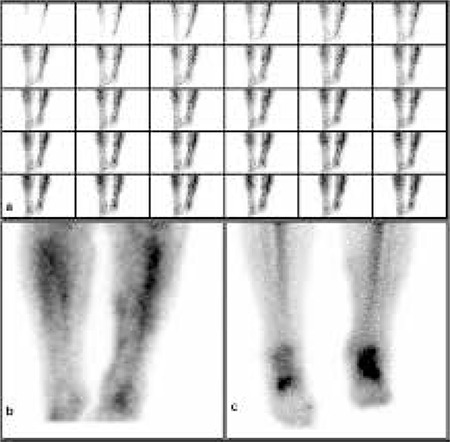
Three phase bone scintigraphy. **a)** Dynamic phase of Tc-99m HDP bone scintigraphy, increased perfusion in left leg and ankle particularly at anterior soft tissue region. **b)** Tc-99m HDP bone scintigraphy, blood pool image of cruris. There is hyperemia in left leg and left ankle compared to their counterparts on the right and also in the medial soft tissue region of left leg, hyperemic foci are observed. **c)** In delayed image there is increased uptake at fracture site of the ankle and moderate increased uptake in soft tissue region.

A 70 year old female patient was referred to our clinic with pre-diagnosis of osteomyelitis or RSD. Her complaints were pain and swelling of left leg and left ankle. Her history revealed that she had broken her left ankle 8 years ago and was operated on her ankle twice, 8 and 4 years ago. Patient was an obese woman and in physical examination her left ankle was mildly hyperemic and edematous, there was no significant rise in heat. She rested heavily on her right leg while she was walking. In the lateral region of her left ankle there was an incision scar and in the 1/3 proximal region of her left leg there were 2 or 3 crusted lesions with about 1 cm diamater. She was on no medication and her laboratory findings were normal. 20 mCi (740 MBq) Tc-99m HDP was administered intravenously and using a large field-of-view gamma camera (Siemens E.CAM/e.soft gamma camera, USA) equipped with a low-energy all purpose collimator, dynamic imaging of both of her ankles was acquired in the anterior projection in 2-s frames for 2 minutes using a matrix size 128x128 in the supine position. After dynamic phase, blood pool image was performed and delayed static and whole body images were obtained 3 hours later. In the dynamic bone scintigraphy there was increased perfusion (Figure 1a) and hyperemia (Figure 1b) in her left ankle and her left leg in the field of view, compared to the counterparts on the right, but there was no significant accumulation of the radiotracer in old fracture region. In the late bone static images, taken at 3 and 24 hours, there was no pathologic radioactivity accumulation consistent with osseos infection, there was increased uptake in fracture site and moderately increased uptake in soft tissue (Figure 1c). We interpreted the increased perfusion and hyperemia at dynamic and blood pool images in her left ankle and her left leg as nonspecific uptake related with venous circulation disorders. But in superficial and Doppler US of patient’s lower extremity, the venous structures were totally normal. We decided to ask further questions about her leg and found out that she had Leech theraphy applied on her leg 4 times within last month, which can explain the increased perfusion and hyperemia in dynamic and blood phases of Tc-99m HDP bone scintigraphy.

## LITERATURE REVIEW AND DISCUSSION

Bone scintigraphy is one of the most useful imaging modalities in Nuclear Medicine clinics to assess osteomyelitis, cellulites, ulceration, RSD, trauma. But the similar appearance of many types of abnormalities have nonspecific scintigraphic findings. Three or four phase bone scan overcomes these problems, so its diagnostic specificity is improved. For instance a combination of focal hyperperfusion, hyperemia and increased bone uptake is diagnostic for acute osteomyelitis, while only increased perfusion and blood pool activity on extraosseous region is seen in cellulitis. Increased radionuclide uptake in early phase of bone scintigraphy which means hyperemia, is affected by extracellullar fluid expansion and enhanced regional vascularity. Differential diagnosis of hyperemia on bone scan can be seen in direct trauma, overuse, electrical injury, soft tissue infection, soft tissue tumor, frostbite, polymyositis, dermatomyositis, active muscular dystrophy, injections (5). Diversity of scintigraphic views that cause hyperemia has increased the importance of questioning the patient.

**Figure 2:**
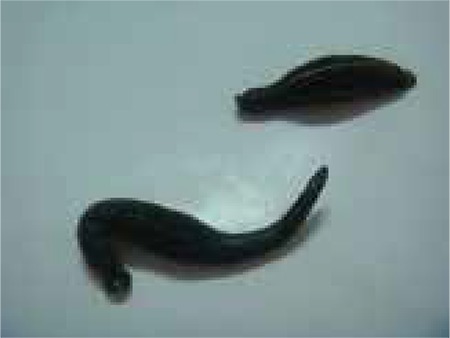
Appearance of Medicinal Leech.

Leech theraphy, which is also known as Hirudotherapy, is termed as Hirudo Medicinalis in historical documents, is nearly 2000 years old. The information that its ectoparasitic properties have been been used for medical treatments can be found in many documents. The oldest documents regarding it are found in Egyptian historical remains that belong to years 1500 BC (6). Nowadays there are many biological, molecular and genetic research on medical use of leech and new species’ (Oligochaeta, Hirudinida, Hirudo) which are increasingly being used in alternative medical treatments (7). Medical leech is a kind of earthworm whose length varies between 5-15 cm (Figure 2). Leech’s saliva has anesthetic, vasodilator, anti-inflammatory, protease inhibitory and anticoagulant (hirudin, saratine) properties and it is thought that it exerts its effect through complex mechanisms. It decreases viscosity by vasodilatation in the applied area, and it is also thought that it exerts its all other effects by inhibiting platelet aggregation, increasing lymph flow and blocking many tissue infection mediators. Although the therapeutic effect of hirudotherapy is not fully understood, it is used in cardiovascular diseases, in many diseases of skeletal system (rheumatological disease, arthritis), in plastic and reconstructive surgery to relieve the local venous congestion after operation and mostly in treatment of pain (4,8). Benefit of hirudotherapy in treatment of pain, particularly in osteoarthritis has been demonstrated with numerous comparative studies that found improvement in symptoms within 4 weeks (9,10). Our patient’s complaints decreased at 3 weeks interval after our scintigraphic study and we believe that she has benefited from this therapy.

## CONCLUSION

In Nuclear Medicine clinics while preparing reports and making interpretation of the scintigraphic findings, the contribution of the history and physical examination of patient is crucial. Depending on dilatory effects of hirudotheraphy in superficial veins, it may lead to an increase in perfusion and hyperemia in blood pool phases of bone scintigraphy, which might be confusing in differential diagnosis.

**Declaration of Interest**

Its no direct or indirect commercial financial incentive associated with publishing the article; There isn’t any source of extra-institutional funding, particularly that provided by commercial sources. All named authors hereby declare that they have no conflicts of interest to disclose.
